# What does One Health want? Feminist, posthuman, and anti-colonial possibilities

**DOI:** 10.1186/s42522-022-00076-9

**Published:** 2023-03-10

**Authors:** Lauren E. Van Patter, Julia Linares-Roake, Andrea V. Breen

**Affiliations:** 1 Department of Clinical Studies, Ontario Veterinary College, University of Guelph, Guelph, ON N1G 2W1 Canada; 2 Department of Family Relations & Applied Nutrition, University of Guelph, Guelph, ON N1G 2W1 Canada

**Keywords:** One Health, Critical social sciences, Critical social theory, Feminist care ethics, Posthuman, Anti-colonial, Multispecies justice

## Abstract

What does One Health want? Despite its touted interdisciplinarity, to date there has been limited engagement with the social sciences and humanities – in particular with streams of critical social theory that enable a response to this question. In this paper we draw on the critical social sciences to consider how One Health is defined, conceptualized, and positioned, and discuss what we see as vital challenges within One Health that both limit its potential for meaningful change and contribute to a potential for ongoing harm – namely, medicalization, anthropocentrism, and colonial-capitalism. We then advance three areas in the critical social sciences that hold potential for addressing these challenges – feminist, posthuman, and anti-colonial approaches. By doing so we seek to encourage a deeper transdisciplinarity within One Health – one that is open to a genuine engagement with insights from critical social theory and a re-orientation towards more creative and radical re-imaginings in the service of wellbeing for diverse peoples, animals, other beings, and the land.

## Introduction

What does One Health want? We are three critical social scientists whose engagements with One Health are part of striving to understand and fulfill our relational responsibilities as settlers working on ancestral Anishinaabe, Hodinöhsö:ni', and Attawandaron territories. We are drawn to this question and to the complexity of answering it. Despite One Health’s touted interdisciplinarity, to date there has been limited engagement with the social sciences and humanities [1], with a particular scarcity of critical social theory informing One Health research and initiatives. In this paper we draw on the critical social sciences to consider how One Health is defined, conceptualized, and positioned, and discuss what we see as vital challenges within One Health that both limit its potential for meaningful change and contribute to a potential for ongoing harm – namely, medicalization, anthropocentrism, and colonial-capitalism. We then advance three areas in the critical social sciences that hold potential for addressing these challenges – feminist, posthuman, and anti-colonial approaches. In so doing our aim is to encourage a deeper transdisciplinarity within One Health, one that is open to genuine engagement with critical social theory and a re-orientation towards more creative and radical re-imaginings in the service of wellbeing for diverse peoples, animals, other beings, and the land.

## Defining One Health: ontology, epistemology, axiology

One Health addresses linkages between animal, human, and environmental health. Aside from this core characteristic, it is challenging to define. Is it an approach? A field of study? It has been described as an ‘epistemic watchword’ – a catalyst serving to bring together diverse actors to engage in interdisciplinary research and practice [2]. With the diversity of interventions in One Health, its specific epistemological, ontological, and axiological^1^ groundings are challenging to pin down [3].

Much of One Health scholarship engages a naturalistic or realist ontology, where what appears to be simply *is*, without sufficient attention to epistemological questions of how knowledge of what ‘is’ comes to be [4]. Knowledge hierarchies privilege dominant science, and especially western ‘biomedical epistemology’ [5, 6]. Knowledges that fall outside of the realm of dominant science are not only devalued, but become unintelligible within a scientistic worldview. As Baquero and colleagues ([7] p5) write:

The universe on the other side of the line [from science] disappears as reality. It becomes non-existent (in the sense of irrelevant and incomprehensible), radically excluded because it is beyond the universe of what the accepted conception of inclusion considers to be its other ([7] p5).

Axiological orientations also remain largely opaque. ‘Health’ is, of course, valued, but in which ways, and for whom? Rock and Degeling ([8] p61) point out that “public health ethics remains weakly articulated with environmental ethics and, to an even lesser extent, with nonhuman animal ethics”. Coghlan et al. ([9] p1) advance that One Health needs to “expand the circle of moral concern beyond a narrow focus on human interests”, and Davis and Sharp ([5] p3) assert that interventions are fundamentally about supporting “the life of wealthy, western, human bodies”. A core issue is that the axiological commitments of One Health interventions are rarely made explicit or interrogated, as the underlying dominant scientific approach remains uncritically grounded in Eurocentric knowledge paradigms which uphold empirical knowledge as objective, universal, value-free, and apolitical. Greater clarity in One Health’s axiological position(s) is an important first step in addressing some of the challenges highlighted below.

## One Health challenges: medicalization, anthropocentrism, colonial-capitalism

### Medicalization

While there is often an acknowledgement that health comprises “the social, political, cultural, economic and spiritual as well as the biomedical”, too often research and interventions in One Health focus narrowly on disease, which “tends to be understood explicitly as a biological matter” ([5] p3). The specificity of this focus neglects dimensions of social wellbeing [10]. Some note the potential for One Health to embrace a more holistic orientation, as opposed to the increasing specialization and compartmentalization of biomedical fields [11]. As Baquero ([12] p9) notes, “[b]iological solutions stripped from the more-than-human social reality will not solve the remarkable challenges posed by mainstream One Health. Indeed, insisting on supposed apolitical and nonideological epidemiologic settings of transmissible and physiopathological processes is part of the problem”.

One Health most often focuses on the negative dimensions of health – on shared vulnerability to disease with a particular emphasis on zoonoses. However, some have advocated that positive dimensions of wellbeing must also be taken into account. Hodgson and Darling ([13] p189) develop the concept “zooeyia”, which is “the positive inverse of zoonosis”. As an example, they discuss the positive wellbeing impacts of the human-animal bond. However, this concept of ‘zooeyia’ is asymmetrically framed around benefits for *humans* alone, leading to the next point.

### Anthropocentrism

One of the frequently cited limitations of One Health is a pervasive anthropocentrism. Linked to human exceptionalism and speciesism, anthropocentrism is a core feature of Eurocentric thought, and central to settler colonialism [14]. As Celermajer et al. ([15] p120) delineate, there are:

three related ideas central to human exceptionalism: a) that humans are physically separate or separable from other species and non-human nature, b) that humans are unique from all other species because they possess minds (or consciousness) and agency and c) that humans are therefore more important than other species.

Anthropocentrism in One Health results in different conceptual modes and ethical frameworks applied to humans versus other-than-human animals. Throughout its history One Health has been “implicitly but firmly devoted to an ethical stance that provided priority to human health over animal welfare” ([11] p186). The realm of the ‘social’ is generally confined to humans, relegating other-than-humans to the space of inert biotic material [12]. Similarly, the ‘public’ in public health only considers humans, not other-than-human community members [16] and assumes “that cared-for others are always human” ([17] p3).

One Health is generally concerned with animal and environmental health not as ends in themselves, but as means to human health. As it is currently theorized and practiced, One Health propagates the inherent anthropocentrism of colonial worldviews by incorporating other species as merely “vectors, reservoirs, or determinants of human health.” ([7] p2). As Kamenshchikova et al. ([18] p310, emphasis added) point out, most often:

animals are understood to be a *resource* for human health; on the other hand, they are considered as potential carriers of diseases. Animal health has to be secured because threats to animal health may transform into health risks *for* *humans*.

In the case of humans, factors apart from physical health or disease alone are more likely to be considered, such as “psychological, emotional, spiritual and economic well-being and socio-political stability” ([19] p53). In other-than-human animals, health tends to remain relegated to physical disease and human use factors, such as “optimal productivity, animal welfare and ethical considerations of animal use” ([19] p53) – a very different set of considerations from those used to characterize human wellbeing. Similarly, ecosystem health takes into account toxins, as well as “plant health, biodiversity, sustainability and resilience of ecosystems” ([19] p53). Discourses around sustainability and resilience are most often framed in anthropocentric terms, as in sustainable human use and resilience to human disturbance, all with a goal of providing for human societies within the context of perpetual growth and the extractivist logics of capital [20].

Discourses of One Health make visible what Agamben [21] terms the ‘Anthropological machine’, where other-than-human life becomes reduced to the status of ‘bios’, ‘bare life’ confined to material bodies who are rendered ‘killable’ and whose deaths are not grievable [5]. As Baquero et al. ([7] p7) note, “[t]he distinction between humans and non-humans is a marginalizing apparatus in the service of domination. It is a central dichotomy of modernity”, and one that remains entrenched within One Health.

### Colonial capitalism

Lainé and Morand ([22] p3) detail the historical emergence of One Health within imperial and colonial projects stemming from a biopolitical need to secure local human and animal health while extracting resources:

a large part of the rhetoric [One Health researchers] use is not new but deeply rooted in the colonial sciences that aimed at developing local societies, their health, and the health of their livestock, as well as their economies by favoring their integration into the Empire market as that time, and to the global market today.

Others note that within One Health research and interventions there can be underlying “hidden assumptions regarding economic growth and liberal governance” ([1] p2). Capital is visible in the commodification of animal bodies, which “transforms the violence perpetrated on sentient beings into procedures to increase production efficiency” ([12] p7). One Health does not challenge this enrollment of living bodies-as-materials into systems of capitalist accumulation. Capitalism is “a shared marginalizing apparatus” – a system, or relation, in which humans, other-than-human animals, and the land are all enmeshed within intersecting relations of power ([7] p2). This brings to mind Wadiwel’s ([23] p147) ‘war on animals’, which “is located upon a violent form of continual appropriation, and an equally violent form of conversion of the lives of animals into value within a human exchange system; property and commodity cohabit as artefacts of war”. It is challenging to disentangle One Health as a biopolical project – making certain populations, namely domesticated farmed animals, live by maintaining health – from a capitalist system which commodifies bodies for profit.

Geopolitically, there is a tendency in One Health to transpose understandings of, and interventions in, health from the global North to the global South and Indigenous Nations. As Baquero et al. ([7] p9) elaborate, “[p]hilanthrocapitalism in health has been a strategy to reinforce colonial epistemology and favor the interests of the global North”, wherein “the only allowed aspiration is to benefit from the epistemological, scientific, and technological transfers of the global North.” ([7] p6). Meanwhile, we cannot ignore that the fundamental assumptions underlying One Health – the interconnection of humans, animals, and the environment – are grounded in unacknowledged Indigenous Ways of Knowing with “[millennia] of successful experiences” ([7] p9). Yet within One Health scholarship, a recent review by Hillier et al. ([24] np) found “[n]o significant connection between One Health and Indigenous knowledges”.

Davis and Sharp ([5] p2) present the following as a synthesis of One Health limitations:

the tendency to universalise western health values (Craddock and Hinchliffe, 2015; Rock, 2017), put humans (and only some humans at that) at the top of a hierarchical structure of health (Brown and Nading, 2019; Hinchliffe, 2015), ignore the social and cultural contexts of health (Woldehanna and Zimicki, 2015), or ignore the political economies that often cause health disparities to begin with (Wallace et al., 2015).

Reflecting these limitations, Baquero et al. ([7] p9) call for a counter-hegemonic ecology of knowledge grounded in ‘post-abyssal thinking’ and global South epistemologies, and constituted by seven actions:

(1) deconstruct apparatuses of marginalization; (2) enrich the ecology of knowledge; (3) build healthy multispecies public policy; (4) create supportive environments; (5) strengthen multispecies community actions; (6) develop individual capabilities in multiple species, and; (7) reorient multispecies health services.

These potentials for alternate knowledges are discussed further below in Sect. 4.3 on anti-colonial opportunities and imperatives in One Health.

## One Health possibilities: feminist, posthuman, anti-colonial approaches

Drawing from theoretical orientations that are central to our own work, we advance that One Health could benefit from deeper engagement with critical social science streams including feminist care ethics, posthumanisms, and anti-colonial approaches. We centre these three for now based on our intellectual groundings, but acknowledge the vital work done in other realms of critical theory, including Critical Disability Studies, Queer Theory, and Black Thought, around which we also hope to see greater engagement in One Health in the future. Each of the following sections ask: *‘what does One Health want, and how might it achieve these ends’*?

### Feminist care ethics

Scholars have recently engaged with the promise of feminism within One Health perspectives [25, 26], arguing that there is an “interconnected and even symbiotic relationship between feminism and One Health” ([26] p1). Human-animal-nature demarcations are bound up in struggles of gendered and racialized oppressions, which necessitate collective and collaborative action attentive to systems of power (e.g. [27, 28, 29, 30, 31, 32]). Adams [33] argues that animal wellbeing cannot be addressed without proper attention to how animal lives are implicated within patriarchal structures. The current separation of gender-species issues, Adams ([33] p 177) argues, “keeps us from making connections”, which impacts scholars in both feminist and Animal Studies arenas. By bringing One Health practitioners into conversation with feminist thought, we can better attend to the complexities and interconnections of power and difference that impact each of the three pillars of One Health. In this section we focus on one strand of feminist thought: feminist care ethics.

Fisher and Tronto ([34] p40, emphasis theirs) define care as “*a species activity that includes everything that we do to maintain, continue, and repair our “world” so that we can live in it as well as possible*”. Feminist care ethicists challenge normative theories which emphasize judgement and reasoning processes as core to moral decision making, and instead suggest decision making processes be guided by emotion, attention, and empathy, which are not universalized principles but instead positioned to value contextual differences within caring relationships [35, 36].

The ethics of care theoretical framework creates space for inter- and intra-species collaboration, with reflection on moral quandaries that come with balancing human, animal, and environmental health [37]. This approach opens up avenues from which to study how and why humans and animals become vulnerable to various health outcomes, and may help practitioners explore the ways in which humans come to care for non-human Others [37, 38, 39]. In doing so, this theory “dislodges the most rigid anthropocentric conceptions of moral considerability and promotes that it is in the interest of humans to maintain ecological and animal health and welfare” ([37] p190).

The ethics of care approach also highlights the negative aspects of care relationships in health and other settings. Because the process of caring for another directly involves power imbalances, there is opportunity to consider the ways in which care itself becomes oppressive [38]. Emphasizing care opens up questions around human and animal suffering (see [37]) and illuminates the ways in which abuse, violence, and care can co-occur within and between species [39].

#### Ecofeminist ethics of care

Ecofeminist theorists have focused on the linkages between environmental degradation, anthropocentrism, and the oppression of individuals across gendered, racialized, and species lines [40, 41]. To think about the growing climate crisis is to think through the structures of *anthroparchy* in the global North; that is, “a complex systems of relations in which the non-human living environment (i.e. organic entities such as animals, plants, soils, seas and contexts for life such as rock and ice scapes) is dominated by human beings as a species” ([40] p5).

Feminist care ethics, and the call to reimagine our ethical commitments, has taken root within ecofeminist practice. Sayers and colleagues ([39] p5) describe such approaches, which “argue our moral conduct towards animals should be guided by affect, sympathetic, and empathetic relations, and the instinct of care, which is forged in close embodied routine practices which involve the so-called mundane practices of everyday women’s work”. The question of whether other-than-human agents have *rights* to welfare is not a central question to ecofeminist care ethicists, who are instead focused on how care manifests between different agents in ways that promote or impact mutual flourishing [41]. In changing the focus, researchers and local communities are able to mobilize daily practices of care across living and non-living agents to address current and future challenges of the Anthropocene [42].

Ecofeminist care theorists also offer insight into the negative implications of more-than-human entanglements. For example, Adams [33] argues that animal suffering is rendered invisible and therefore accepted by society so long as said suffering directly benefits human progress. Animal suffering can – and often does – gain visibility only *when* we attribute agency to an individual animal [33, 39]. Thinking with care allows researchers to situate multiple interests – human and nonhuman alike – within moral issues, thus rendering visible the suffering of those often ignored in dominant narratives [38]. By focusing on contextual relationships rather than strict claims around how to create livable worlds, ecofeminist care ethics allows research to take up recent calls within One Health literature to take seriously local knowledges and lived experiences (see [43]).

From a feminist care perspective, does One Health want to continue to perpetuate a single narrative about what it means to have good health, or will it allow care to guide it through the messiness of the complex, interconnected world in which we live?

### Posthumanism(s)

Posthumanism(s) – in their various formations – speak back to humanist paradigms: the dominant systems of thought underlying Eurocentric science, humanities, legal orders, etc., that define subjecthood or personhood around particular ideals of the human. The posthumanism proposed here is of the critical strain, which aims to analyse intersecting domains of oppression across species borders while also necessarily being occupied with new and transformative futures beyond humanism [44]. These understandings are embroiled with definitions and classifications of species, as well as gender, race, ability, and other markers, as the ‘Subject’ of enlightenment humanism – and still dominant benchmark of personhood – is the white, able-body-minded male. Posthumanism challenges this understanding, “rejecting the presumably autonomous human body of Enlightenment thought” ([45] p453), through avenues that include acknowledging our microbial co-constitution, and the reality of, and value in, considering the self-hood or experiences of other-than-human beings.

There has been limited engagement with posthumanism(s) within One Health. Kirk et al. ([46] p475) draw on emergent work in the medical humanities and more-than-human/multispecies studies to articulate the value of a ‘multispecies medicine’ which recognizes that “if being human is a process of becoming together, by extension being well becomes a process of being well together”.

Posthuman approaches can contribute to One Health by disrupting its underlying anthropocentrism, in particular “paying critical attention to the entanglement of human and animal, to their mutual becoming and ‘shared suffering’ in the context of capitalism and post-colonial encounters” ([5] p4). As Baquero ([12] p8) notes, we need to take seriously a “more-than-human social determination of health”, where other-than-human species are also encountered as bearers of health, or as beings whose wellbeing matters. To do so, we need inquiries that acknowledge animals’ “contribution to both sociality and the emergence of new and dynamic social environments” ([47] p561). There are two avenues in particular where posthuman approaches have been, or could be, mobilized within One Health: Multispecies publics & solidarity; and Multispecies justice.

#### Multispecies publics & solidarity

Posthumanism has already entered the dialogue around One Health in the work of anthropologist and social worker Melanie Rock [8, 16, 17, 48]. Rock challenges the inherent anthropocentrism in the fields of public health and health promotion, querying who is the ‘public’ in public health, and what happens when we do away with the assumption that it is inevitably only members of the human species, recognizing that “people’s efforts to care for non-human others are highly relevant to public health” ([8] p61). Rock and Blue [17] advance that we need a posthuman approach to health promotion, recognizing the important role other-than-human animals play in public health and individual wellbeing. The conceptualisation of ‘multispecies publics’ makes visible the ways in which “the emergence and evolution of publics depend on multi-species entanglements” ([17] p2), for instance in the case of companion animals in disaster response, recovery, and preparedness.

Rock and Degeling ([8] p62) delineate a ‘more-than-human solidarity’, which they define as “human activity directed towards carrying costs and making tradeoffs of various kinds with the intent of assisting others, whenever cared-for others include nonhuman animals, plants, or places”. Their focus is on companion animals in particular, as the often cared-for other-than-humans in many individuals’ lives, but the concept could be more broadly extended to other beings and the land. More-than-human solidarity provides a grounding for ethical dialogues that acknowledge the interconnectedness of wellbeing, while delivering an actionable, rather than merely conceptual, path.

#### Multispecies justice

There are many ways that intersectional multispecies justice approaches can bolster One Health. For instance, Kirk et al. ([46] p78) argue that it would be helpful to bring into dialogue insights from Animal Studies and Critical Disability Studies to explore “questions concerning shared health, vulnerably, interdependencies and social justice”. Baquero ([12] p3) notes the importance of attending to intersectionality in the context of One Health, as biopolitical apparatuses that create suffering “are constituents of speciesist, racist, ethnic, class, gender, capacity, and geographic marginalization”.

There are challenges to delineating and mobilizing interventions aimed at multispecies justice. These include the challenges of imaging: what would a “non-fearful, non-bordered, non-masterful, nonanthropocentric approach be” ([27] p25)? Many Animal Studies scholars and activists have recognized the challenges of bringing other species into social justice debates. This cannot be dismissed, but rather we need to find ways to combat “entrenched dehumanization while acknowledging the real, embodied hardship of social inequalities, multidimensional poverty, and pervasive injustices in all societies” ([15] p133). Another challenge is how to determine other-than-human interests “without assimilating them into our own forms of understanding and being” ([49] p480).

In order to address such challenges, our approach needs to be a difference-oriented justice with a focus on shared vulnerabilities [31, 49, 50, 51]. We need to “consider human and nonhuman health, welfare, and rights (including the right to health) holistically and structurally” ([52] p). And we need new forms of knowledge, as “the colonial imperative to assimilate other lifeworlds through full knowledge as mastery eliminates the possibility for multispecies justice” ([49] p492). Our approach needs to be “self-consciously decolonizing and deconstructive of liberal hegemony” ([15] p129), with meaningful, non-appropriative engagements with Indigenous knowledges that do not divorce teachings from the land, language, and culture that gives them meaning.

Srinivasan [53] writes that multispecies justice requires a reanimalization of the human, wherein we move away from ‘zoöpolitical exceptionalism’ which sees human wellbeing as something to be achieved by overcoming nature through development. Only when we truly understand ourselves as part of nature, as one species among many, can we begin the task of redistributing the risks of life on this planet across human and nonhuman Others to work against marginalization. Emel and Nirmal ([54] p34) advance that we must:

Examine multiple oppressions with great care without privileging one over another. Employ democratic, pluralistic and decolonial methods, ideas and practices when engaging with complex and emergent political ecologies. Engage with multispecies justice not just for the sake of developing further theoretical analyses, but with a concerted focus on policy and institutional change.

The potentials of multispecies justice are beginning to be recognized outside of academic circles; for instance in the Phoenix Zones Initiative [55]’s Just One Health, which recognizes and advances the “interconnected rights, health, and wellbeing of vulnerable, people, animals, and the planet”.

Finally, posthuman approaches open space to reconsider assumptions around knowledge and knowing, namely: who is a knower? Are animals’ knowledges of their own health needs and connections a relevant consideration within One Health? Lainé and Morand [22] propose that through the use of multispecies ethnographic methodologies, social science researchers can gain insights into the connections between local knowledge, animal knowledges, and One Health concerns like epidemic outbreaks. Within some cultures, including those of many Indigenous peoples, non-human animals, plants, and the land are positioned as knowers (e.g. [56]).

From a posthuman perspective, does One Health want to continue serving the narrow interests of (some) humans, or is it willing to broaden its focus, value structures, and approaches to consider the lives, interests, and wellbeing of a diverse array of beings with whom we share the planet?

### Anti-colonial

Poor health outcomes (human and animal) and environmental degradation cannot be separated from the structural violences of slavery, environmental racism, patriarchy, and ongoing colonialism [57, 58]. The imposition of Eurocentric notions of human-being—which emphasize humans as separate from non-human animals and the land— is foundational to violence against animals, the land, and also to the marginalization and oppression of certain groups of human beings who have been subject to logics of dehumanization, including Black and Indigenous peoples [14, 59, 60] and persons with disabilities [61].

As Belcourt ([59] p21) argues in *an Indigenous Critique of Critical Animal Studies*, “we cannot address animal oppression and liberation without beginning from an understanding that settler colonialism and white supremacy are the bedrock of much of the structural violence that unfolds on occupied Indigenous territory”. Darren Chang writes ([62] p38), “all efforts to defend wild animals and the ecological environment they depend on will most likely fail if settler-colonial capitalist processes of dispossessions and destructions are not dismantled”. One Health researchers and practitioners need to be aware of the ways in which scientific knowledge and the academy extract and appropriate Indigenous knowledges, while simultaneously enacting epistemic violence [12, 58]. Researchers must be aware of how Indigenous communities share experiences of “genocidal and assimilative practices and policies carried out by settler-colonial states [as well as] differences in diverse cultural and historical backgrounds of Indigenous peoples” ([62] p29). Indigenous Nations are diverse and the particulars of land and relations matter when considering health and wellbeing.

There is a need for “leadership shift” and “knowledge shift” [57] with Indigenous peoples and communities leading processes of knowledge generation and disruptions to the flow of knowledge so that it moves not only from the northern hemisphere to southern and from settler colonial institutions like universities to communities, but in multiple directions, as well as in multiple languages (beyond English), and through multiple Ways of Knowing (beyond dominant science). A necessary component of this “knowledge shift” is creating space for animal agency [62] and multispecies knowledges. It requires a “decolonial politics that conceptualizes animals as kin who co-produce a way of life that engenders care rather than and contra to suffering” ([59], p24-25).

There is also a need for a “paradigm shift” [57], with “individuals and institutions acknowledging that disease cannot be extracted or isolated from broader systems of coloniality” and “changing who sits at the table and rebuilding parts of the table itself” ([57] p3). These shifts require awareness of the ways in which white supremacy and white saviourism operate in systems of knowledge production and resultant interventions. As la paperson ([63] p9-10) argues, it requires us to examine our knowledge systems as “machinery commissioned to actualize imperialist dreams of a settled world” and “desire[s] to humanize the world, which is a more genteel way to colonize a world that is so much more than human”. Another way is possible: but this requires “the rematriation of land, the regeneration of relations, and the forwarding of Indigenous and Black and queer futures—a process that requires countering what power seems to be up to” ([63] p10). The critical social sciences allow us to peer behind the curtain at power relations, to make visible and evaluate both the benefits and potential harms of what One Health may be up to.

From an anti-colonial perspective, what does One Health want its role to be in relation to ongoing settler colonialism and white supremacy? Does One Health want to contribute to ongoing colonial violence or is it willing to critically examine its practices, structures, and values and contribute to an anti-colonial world where health is fully realized for Indigenous peoples, other-than-human animals, and the land?

## Conclusions

This paper explores what we see as some of the key challenges and opportunities of One Health as a collaborative and interdisciplinary orientation to research, focusing on the value of deeper engagement with critical social theory. Others have noted the need for more meaningful engagement between One Health and the social sciences (e.g. [1, 43]), highlighting, for instance, the importance of generating greater understandings of the ways in which health and wellbeing are related to “differential positioning or placement in social settings and economic markets” ([1] p2).

In this paper, drawing from our own intellectual groundings, we advance the value of feminist ethics of care, posthuman theories, and anti-colonial approaches. We acknowledge there are many additional areas of critical theory of vital importance and with potential value to One Health which are also in need of greater engagement, including Critical Disability Studies, Queer Theory, and Black Thought.

As a final thought, we encourage others to continue exploring the question, “what does One Health want?” Does it take as its key project improving the world as it is, within the frameworks of existing modes of production and relation? Or is there space to fundamentally shift the way we live, work, and relate to other beings and the land so as to address key intersecting health challenges and promote more-than-human wellbeing, care, and justice? We feel that to date the focus has been the former, and assert that this paradigm does not hold promising potential for addressing the root causes of wellbeing concerns and inequities across species – or indeed, across individuals and groups within our own species. We need to find ways of pushing One Health towards the latter, and advance the value of engaging critical approaches from the social sciences – such as feminist, posthuman, and anti-colonial traditions – in nurturing this change.

## Data Availability

Not applicable.

## Footnotes

1 For definitions of ontology, epistemology, and their importance to natural scientists, see [64]; for a discussion of axiology in interdisciplinary research, see [65].

## References

[1] Craddock S, Hinchliffe S (2015). One world, one health? Social science engagements with the one health agenda. Soc Sci Med.

[2] Michalon J (2020). Accounting for One Health: Insights from the social sciences. Parasite.

[3] Wolf M (2015). Is there really such a thing as “one health”? Thinking about a more than human world from the perspective of cultural anthropology. Soc Sci Med.

[4] Hinchliffe S (2015). More than one world, more than one health: Re-configuring interspecies health. Soc Sci Med.

[5] Davis A, Sharp J (2020). Rethinking One Health: Emergent human, animal and environmental assemblages. Soc Sci Med.

[6] Estebanez J, Boireau P (2022). One Health: A social science discussion of a global agenda. Parasite.

[7] Baquero OS, Benavidez Fernández MN, Acero AM (2021). From modern planetary health to decolonial promotion of One Health of peripheries. Front Public Health.

[8] Rock MJ, Degeling C (2015). Public health ethics and more-than-human solidarity. Soc Sci Med.

[9] Coghlan S, Coghlan BJ, Capon A, Singer P (2021). A bolder One Health: expanding the moral circle to optimize health for all. One Health Outlook.

[10] Degeling C, Rock M (2020). Qualitative research for One Health: From methodological principles to impactful applications. Front Vet Sci.

[11] Beever J, Morar N (2019). The epistemic and ethical onus of ‘One Health’. Bioethics.

[12] Baquero OS (2021). One Health of peripheries: Biopolitics, social determination, and field of praxis. Front Public Health.

[13] Hodgson K, Darling M (2011). Zooeyia: an essential component of “One Health”. Can Vet J.

[14] Belcourt BR (2014). Animal bodies, colonial subjects: (Re)Locating animality in decolonial thought. Societies.

[15] Celermajer D, Schlosberg D, Rickards L, Stewart-Harawira M, Thaler M, Tschakert P (2021). Multispecies justice: theories, challenges, and a research agenda for environmental politics. Env Polit.

[16] Rock MJ (2017). Who or what is ‘the public’ in critical public health? Reflections on posthumanism and anthropological engagements with One Health. Crit Public Health.

[17] Rock M, Blue G (2020). Healthy publics as multi-species matters: solidarity with people’s pets in One Health promotion. Humanit Soc Sci Commun.

[18] Kamenshchikova A, Wolffs PFG, Hoebe CJPA, Horstman K (2021). Anthropocentric framings of One Health: An analysis of international antimicrobial resistance policy documents. Crit Public Health.

[19] Lapinski MK, Funk JA, Moccia LT (2015). Recommendations for the role of social science research in One Health. Soc Sci Med.

[20] Adelman S, French D, Kotze L (2018). The Sustainable Development Goals, Anthropocentrism and neoliberalism. Sustainable Development Goals : Law, Theory and Implementation.

[21] Agamben G. The Open: Man and Animal. Stanford: Stanford University Press; 2004.

[22] Lainé N, Morand S (2020). Linking humans, their animals, and the environment again : a decolonized and more-than-human approach to “One Health”. Parasite.

[23] Wadiewl D. The War Against Animals. Leiden: Brill; 2015.

[24] Hillier SA, Taleb A, Chaccour E, Aenishaenslin C. Examining the concept of One Health for indigenous communities: A systematic review. One Health. 2021;12:100248. 10.1016/j.onehlt.2021.100248 PMC8066803 33912647

[25] Heidari S, Doyle H (2020). An invitation to a feminist approach to global health data. Health Hum Rights.

[26] Hardy E, Standley CJ (2022). Identifying intersectional feminist principles in the One Health framework. One Health.

[27] Emel J, Wolch J, Emel J (1998). Are you man enough, big and bad enough? Ecofeminism and wolf eradication in the USA. Animal Geographies: Place, Politics, and Identity in Nature-Culture Borderlands.

[28] Hovorka AJ (2015). The gender, place and culture Jan Monk Distinguished annual lecture: Feminism and animals: exploring interspecies relations through intersectionality, performativity and standpoint. Gend Place Cult.

[29] Kim CJ. Dangerous Crossings. Cambridge: Cambridge University Press; 2015.

[30] McCubbin SG, van Patter LE (2021). Trophy Hunters & Crazy Cat Ladies: exploring cats and conservation in North America and Southern Africa through intersectionality. Gend Place Cult.

[31] Plumwood V. Feminism and the Mastery of Nature. Abingdon: Routledge; 2002.

[32] Seager J (2003). Pepperoni or Broccoli? On the cutting wedge of feminist environmentalism. Gend Place Cult.

[33] Adams C, Donovan J, Adams C (1995). Caring about suffering: A feminist exploration. Beyond animal rights: A feminist caring ethic for the treatment of animals.

[34] Fisher B, Tronto J, Abel E, Nelson M (1990). Toward a feminist theory of caring. Circles of care: Work and identity in women’s live.

[35] Collins S. The Core of Care Ethics. London: Palgrave Macmillan; 2015. p. 1–81.

[36] Pettersen T (2008). Comprehending Care: Problems and Possibilities in The Ethics of Care.

[37] Anthony R, de Paula VA (2022). One Health animal disaster management: An ethics of care approach. J Appl Anim Welfare Sci.

[38] de la Bellacsa MP. Matters of Care: Speculative Ethics in More than Human Worlds. Minneapolis: University of Minnesota Press; 2017.

[39] Sayers J, Forrest R, Pearson M (2022). Furry families: Ethical entanglements through more-than-human domestic dramas. Sociol Res Online.

[40] Cudworth E (2005). Developing Ecofeminist Theory.

[41] Curtin D (1991). Toward an ecological ethic of care. Hypatia.

[42] Serafini P (2021). A decolonial, ecofeminist ethic of care. Soc Anthropol.

[43] Steffens T, Finnis E (2022). Context matters: Leveraging anthropology within one health. One Health.

[44] Cudworth E, Hobden S. The Emancipatory Project of Posthumanism. Abingdon: Routledge; 2017.

[45] Cole L (2021). Aliens, plagues, One Health, and the medical posthumanities. Configurations.

[46] Kirk RGW, Pemberton N, Quick T (2019). Being well together? Promoting health and well-being through more than human collaboration and companionship. Med Humanit.

[47] Chalmers D, Dell CA (2015). Applying One Health to the study of animal-assisted interventions. EcoHealth.

[48] Rock M (2013). Pet bylaws and posthumanist health promotion: a case study of urban policy. Crit Public Health.

[49] Celermajer D, Chatterjee S, Cochrane A, Fishel S, Neimanis A, O’Brien A (2020). Justice through a multispecies lens. Contemporary Political Theory.

[50] Derrida J. The Animal that Therefore I Am. Mallet ML, Wills D, editors. New York: Fordham Univ Pres;2008.

[51] Srinivasan K, Kasturirangan R (2016). Political ecology, development, and human exceptionalism. Geoforum.

[52] Sellars L, Bernotas K, Sebo J (2021). One Health, COVID-19, and a right to health for human and nonhuman animals. Health Hum Rights.

[53] Srinivasan K (2022). Re-animalising wellbeing: Multispecies justice after development. Sociol Rev.

[54] Emel J, Nirmal P, Horvoka A, McCubbin S, van Patter L (2021). A feminist research agenda for multispecies justice. A Research Agenda for Animal Geographies.

[55] Phoenix Zones Initiative. https://www.phoenixzonesinitiative.org/what-is-just-one-health/. Accessed 07 May 2022

[56] Watts V. Indigenous place-thought and agency amongst humans and non humans (First Woman and Sky Woman go on a European world tour!). Decolonization Indigeneity Educ Soc. 2013;2(1).

[57] Büyüm AM, Kenney C, Koris A, Mkumba L, Raveendran Y (2020). Decolonising global health: if not now, when?. BMJ Glob Health.

[58] Liboiron M (2021). Pollution Is Colonialism.

[59] Belcourt BR, Montford K, Taylor C (2020). An Indigenous critique of Critical Animal Studies. Colonialism and Animality.

[60] Bennett J. Being property once myself: Blackness and the end of man. Harvard University Press; 2020.

[61] Taylor S (2017). Beasts of burden: Animal and disability liberation.

[62] Chang D, Montford K, Taylor C (2020). Tensions in contemporary Indigenous and animal advocacy struggles. Colonialism and Animality.

[63] lapaperson (2017). A third universe is possible.

[64] Moon K, Blackman D (2014). A guide to understanding social science research for natural scientists. Conserv biol.

[65] Biddle C, Schafft KA (2015). Axiology and anomaly in the practice of mixed methods work: Pragmatism, valuation, and the transformative paradigm. J Mix Methods Res.

